# The cascade of care following community-based detection of HIV in sub-Saharan Africa – A systematic review with 90-90-90 targets in sight

**DOI:** 10.1371/journal.pone.0200737

**Published:** 2018-07-27

**Authors:** Kalpana Sabapathy, Bernadette Hensen, Olivia Varsaneux, Sian Floyd, Sarah Fidler, Richard Hayes

**Affiliations:** 1 London School of Hygiene and Tropical Medicine, London, United Kingdom; 2 Imperial College London, London, United Kingdom; National and Kapodistrian University of Athens, GREECE

## Abstract

**Introduction:**

We aimed to establish how effective community-based HIV testing services (HTS), including home and community location based (non-health facility) HIV testing services (HB-/CLB-HTS), are in improving care in sub-Saharan Africa (SSA), with a view to achieving the 90-90-90 targets.

**Methods:**

We conducted a systematic review of published literature from 2007–17 which reported on the proportion of individuals who link-to-care and/or initiate ART after detection with HIV through community-based testing. A meta-analysis was deemed inappropriate due to heterogeneity in reporting.

**Results and discussion:**

Twenty-five care cascades from 6 SSA countries were examined in the final review– 15 HB-HTS, 8 CLB-HTS, 2 combined HB-/CLB-HTS. Proportions linked-to-care over 1–12 months ranged from 14–96% for HB-HTS and 10–79% for CLB-HTS, with most studies reporting outcomes over short periods (3 months). Fewer studies reported ART-related outcomes following community-based testing and most of these studies included <50 HIV-positive individuals. Proportions initiating ART ranged from 23–93%. One study reported retention on ART (76% 6 months after initiation). Viral suppression 3–12 months following ART initiation was 77–85% in three studies which reported this.

There was variability in definitions of outcomes, numerators/denominators and observation periods. Outcomes varied between studies even for similar time-points since HTS. The methodological inconsistencies hamper comparisons. Previously diagnosed individuals appear more likely to link-to-care than those who reported being newly-diagnosed. It appears that individuals diagnosed in the community need time before they are ready to link-to-care/initiate ART. Point-of-care (POC) CD4-counts at the time of HTS did not achieve higher proportions linking-to-care or initiating ART. Similarly, follow-up visits to HIV-positive individuals did not appear to enhance linkage to care overall.

**Conclusion:**

This systematic review summarises the available data on linkage to care/ART initiation following community-based detection of HIV, to help researchers and policy makers evaluate findings. The available evidence suggests that different approaches to community-based HTS including HB-HTS and CLB-HTS, are equally effective in achieving linkage to care and ART initiation among those detected. Engagement and support for newly diagnosed individuals may be key to achieving all three UNAIDS 90-90-90 targets. We also recommend that standardised measures of reporting of steps on the cascade of care are needed, to measure progress against targets and compare across settings.

## Introduction

UNAIDS 90-90-90 targets aim to ensure that by 2020, 90% of all people living with HIV will know their HIV status (first-90); 90% of all people with diagnosed HIV infection will receive sustained antiretroviral therapy (second-90); 90% of all people receiving antiretroviral therapy will have viral suppression (third-90). In combination, achieving these three targets would lead to 73% of PLWH being virally suppressed, and mathematical models suggest this would enable the “ending” of the AIDS epidemic by 2030.[1] Using out-of-facility, community-based approaches to increase knowledge of HIV status in sub-Saharan Africa (SSA) towards achieving the first-90, now seems ever more attainable.[2–5] These approaches detect infected individuals earlier in the course of infection.[3] The benefits of early treatment for those who are infected, and for the prevention of onward transmission, are now firmly established.[6–8] The benefits may conversely pose challenges for timely linkage to care. Individuals who feel well may not be ready to access care at health facilities even when provided with a diagnosis. While community-based approaches of HIV-testing reduce barriers for testing, the challenges associated with health facilities remain and individuals identified by community-based HTS may be less likely and/or take longer to link-to-care.

Linkage to care should result ultimately in viral suppression among people living with HIV (PLWH). The 90-90-90 targets provide a standard against which performance can be measured. This systematic review examines published evidence from sub-Saharan Africa on linkage to care, initiation of ART and retention/viral suppression if reported, following out-of-facility community-based detection of HIV, with the 90-90-90 targets in mind—in particular the second- and third-90s.

## Methods

We conducted a systematic review according to the criteria in PRISMA guidelines [9] (S1 File PRISMA Checklist) and devised a pre-defined search protocol. Our primary objective was to evaluate the proportion of individuals, detected with HIV through community-based testing, who i.link into HIV care, ii.start ART, iii. are virally suppressed. We also sought to identify if there were differences in linkage and ART initiation based on the HIV testing approach and methods used to enhance engagement with the cascade of care. We use the term HIV detection to refer to HIV diagnosis through community-based HIV testing services (HTS) or self-report of known HIV-positive status at the time of HTS in the community.

### Search strategy

We summarised studies that described the cascade of care following HIV detection through community-based approaches (namely home based HTS (HB-HTS) or HTS (at community locations which use mobile units, temporary structures or provide HTS in existing non-health facility community venues ie community location based HTS (CLB-HTS)), in SSA. We searched Pubmed, Embase and Global Health electronic databases. We developed a broad compound search strategy that combined terms for “home based”, “mobile”, “community”, “work-place”, “school-based”, “self-testing” “HIV” and “voluntary counselling and testing” (S2 File Search Terms). We also manually searched the bibliographies of relevant articles.

Inclusion criteria for the review were studies which reported the proportion of individuals, detected with HIV through community-based testing, who link into HIV care and/or start ART, in SSA which were published between January 2007 and May 2017—examining data from the last decade to reflect the period during which community-based testing has become more widespread and to maximise relevance to current practice. We allowed observational studies with data from routine service delivery, cross-sectional, case-control and cohort studies, as well as randomised controlled trials (including cluster randomised trials). We excluded data on HIV testing in health-care facilities (HCFs) (or satellite sites of HCFs), or treatment initiation in the household, as our primary focus was on linkage to care to receive services (including ART initiation) at HCFs. We also excluded reports that pooled data from previously published studies to avoid duplication. Where there was substantial overlap of study subjects in more than one paper, we included the paper with the most complete information. Our search was limited to English language peer-reviewed journal articles, with no age restrictions for participants. We excluded conference abstracts.

### Qualitative analysis of studies

Eligibility of articles was determined independently by two investigators (KS and OV). Using a standardised data-extraction form (KS and BH) independently extracted data on study characteristics and outcomes, with input from OV. Any disagreements were resolved by consensus. Potential citations (published articles and conference papers after removal of duplicates) which were identified from the search strategy were reviewed for suitability. Citations which were on conference abstracts or were unrelated to community approaches of HIV testing in sub-Saharan Africa—for instance studies from other countries, laboratory studies or articles which were not reporting primary research—were excluded. Titles and abstracts were then examined and excluded if they did not report HIV testing, linkage to care or were on facility based HIV testing. Full text articles were then reviewed for full inclusion and exclusion criteria as detailed above.

Markers of study quality were examined [9] and strengths and limitations of the studies are presented along with propensity for bias. The latter was examined using a modified Cochrane Collaboration approach for assessing risk of bias.[10] We focused on three main domains in relation to our study objectives—selection bias (eg whether those who were already in care were excluded from linkage to care outcome calculations), outcome ascertainment (eg whether objective measures such as clinic records were sought to determine LTC) and attrition (with a cut-off of ≥20% loss-to-follow up as high attrition)—before summarising if the risk of bias in a study was low, medium or high overall. Studies were not excluded for quality reasons using formal criteria for reporting scientific data, not least because a large proportion of the available data came from operational delivery of HTC services and authors presented data as were available from the programmes.

Ethical approval was not required as only published literature was included for review.

### Data synthesis and analysis

If studies reported different approaches to testing (eg by study arm) we reported linkage outcomes by modality (eg CLB-HTS or HB-HTS) where possible. We calculated the proportion of individuals: (i) linked-to-care and (ii) initiated on ART and explored time to linkage to care and ART initiation. We used information as was available from the papers reviewed but note here that denominators often differed between studies, especially for ART initiation with some authors drawing from all those identified HIV-positive while others limited their denominator to those who presented for care, for instance. Table 1 therefore presents in detail the exact populations used in the numerator and denominator to calculate proportions for (i). and (ii). above. Finally, we summarised retention on ART among those who initiated and extracted data on viral suppression, if studies reported this.

**Table 1:** Key characteristics of included studies.

Author, Year, Country, Rural/ Urban	Testing approach Intervention(s) to enhance Linkage To Care (LTC)	Proportion HIV+ (%)*	Number HIV+		Numerator for % LTC	Denominator for % LTC	Proportion LTC % (n/N)	Numerator for % initiated ART	Denominator for % initiated ART	Proportion initiated ART % (n/N)
			Newly identified HIV+	Known HIV+, not in care/ on ART						
**Home-based HTS (HB-HTS)**										
**Barnabas, 2014**^1^ **[29]** South Africa & Uganda, Rural & peri-urban	Door-to-door HB-HTS study; POC CD4-count, Written referral, Lay counsellor FU	19	229	152	n visiting an HIV clinic	N newly diagnosed or known HIV+ not on ART	96% (367/381)	n initiated ART	N newly diagnosed or known HIV+ not on ART & CD4 <350/cc^3^	76% (94/123)
**Dalal, 2013 [14]** Kenya, Rural + urban	Door-to-door HB-HTS implementation; Written referral, Lay counsellor FU	16	1839	Not reported	n accessing patient support centre	N newly diagnosed	47% (454/958)	n initiated ART	N newly diagnosed adults & CD4 <250/cc^3^	34% 43/125
**Genberg, 2015 [15]** Kenya, Rural	Door-to-door HB-HTS implementation; Verbal referral	11	1360	344	n having clinical encounter with HIV care provider	N newly diagnosed or known HIV+ not in care	14% (243/1704)	n initiated ART	N newly diagnosed, eligible and LTC ^2^	85% (78/92)
									N known HIV+ not on ART ^2^	53% (18/34)
**Iwuji, 2016 [31]** South Africa, Rural	Door-to-door HB-HTS within cluster randomised trial; Referral; FU if failure to link (home visit or telephone), ART for all PLWH in intervention arm	31	264 (interv.) + 310 (control arm)	349 (interv.) + 416 (control arm)	n visiting clinic (according to clinic database)	N newly diagnosed or known HIV+ not in care	63% (191/305) (interv.)	n initiated ART	All PLWH irrespective of CD4 count)	89% (194/218) (interv.)
							64% (185/291) (control arm)			42% (83/196) (control arm)
**Labhardt, 2014 [11]** Lesotho, Rural	Door-to-door HB-HTS and multi-disease services (within a cluster randomised trial); Referral only	4	39	Not reported	n linked to care	N newly diagnosed	26% (10/39)	Not reported	Not reported	Not reported
**MacKellar, 2016 [17]** Swaziland, Rural + urban	Door-to-door HB-HTS national campaign; Written referral, Text/call reminder, Call 3d after missed visit / FU visit	Not reported	850	Not reported	n received CD4 count result or WHO staged	N newly diagnosed	27% (209/788)	Not reported	Not reported	Not reported
**Maman, 2016 [32]** Malawi, Rural	Door-to-door HTS within population cross-sectional survey; POC CD4-count, Written referral	17	282	Not reported	n visiting clinic (according to clinic database)	N newly diagnosed	47% (115/244)	Not reported	Not reported	Not reported
**Medley, 2013 [19]** Kenya, Rural	Door-to-door HTS within demographic surveillance; Written referral, Peer educator FU	11	923	Not reported	n currently attending to HIV clinical care	N adults tested HIV+	42% (312/737)	n on ART	N adults tested HIV+ and LTC ^2^	26% (80/312)
**Naik, 2015 [27]** South Africa, Rural	Door-to-door HB-HTS study; Written referral (for CD4-count at clinic)	10	274	Not reported	n linked to care	N clients tested HIV+ not already in pre-ART or ART care	76% (273/359)	Not reported	Not reported	Not reported
**Tumwebaze, 2012**^1^ **[20]** Uganda, Rural & peri-urban	Door-to-door HB-HTS study; POC CD4-count, Written referral	10	77	36	n visiting an HIV clinic	N newly diagnosed or known HIV+ but not on ART	85% (96/113)	n initiated ART	N newly diagnosed or known HIV-positive not on ART&CD4 <250/cc^3^	71% (15/21)
**van Rooyen, 2014 [21]** South Africa, Rural	Door-to-door HB-HTS study; POC CD4-count, Written referral	30	73	64	n visited HIV clinic	N adults newly diagnosed or known HIV+ but not on ART	96% (131/137)	n initiated ART	N newly diagnosed &CD4 <350/cc^3^ and LTC	54% (19/35)
									N known HIV+, not on ART & CD4 <350/cc^3^ and LTC	65% (17/26)
**MacPherson, 2014 [18]** Malawi, Urban	Study involving self-testing with oral test kits offered to household members (within a cluster randomised trial); Referral only	Not reported	278	Not reported	Not reported	Not reported	Not reported	n initiated ART	N reporting HIV+ self-test result & CD4 <350/cc^3^ and LTC	23% (63/376)
**Shapiro, 2012 [12]** South Africa, urban	Index case (TB patients) driven HB-HTS study; Referral only (letter for ART eligible/verbal for non-eligible)	15	Not reported	Not reported	Not reported	Not reported	Not reported	n initiated ART	N HIV+ household contacts of a TB index case & CD4 <250/cc^3^	41% (13/32)
**Shapiro, 2012 [12]** South Africa, urban	Randomly selected household HB-HTS study; Interventions as above	11	Not reported	Not reported	Not reported	Not reported	Not reported	n initiated ART	N HIV+ non- contact participants with CD4 <250/cc^3^	53% (10/19)
**Velen, 2016 [33]** South Africa, Rural & urban	Nested cohort study within control arm of cluster randomised trial; Written referral	14	26	108	n newly diagnosed and reporting entry into care	N newly diagnosed	35% (8/23)	Not reported	Not reported	Not reported
**Community location based- (CLB-) and HB-HTS**										
**Barnabas, 2016 [28]** South Africa & Uganda, Rural	Door-to-door HB-HTS & HTS in mobile units (LTC assessed within factorial design randomised controlled trial); Randomised comparison of POC CD4-count vs clinic CD4-count & Randomised comparison of lay counsellor FU vs lay counsellor clinic facilitation vs referral only	15	992	333	n visiting an HIV clinic	N newly diagnosed & known HIV+ not on ART (Lay counsellor FU arm)	93% (419/449)	n initiated ART	N newly diagnosed & known HIV+ not on ART (Lay counsellor FU arm)	41% (185/449)
						N newly diagnosed & known HIV+ not on ART (Clinic facilitation arm)	98% (421/431)		N newly diagnosed & known HIV+ not on ART (Clinic facilitation arm)	37% (161/431)
						N newly diagnosed & known HIV+ not on ART (Referral only arm)	89% (378/423)		N newly diagnosed & known HIV+ not on ART (Referral only arm)	34% (142/423)
**Parker, 2015 [30]** Swaziland, Rural	Door-to-door HB-HTS implementation; Written referral, Phone reminder, Phone/FU for missed visit	4	242	12	n registered in pre-ART care	N newly diagnosed	34% (135/398)	n initiated ART	N newly diagnosed & CD4 <350/cc^3^ and LTC	52% (22/42)
	HTS in tents at several community locations; Interventions as above	5	96	12						
**Community location based HTS (CLB-HTS)**										
**Bassett, 2015 [23]** South Africa, Urban	Mobile units at taxi stands, markets, and sporting grounds; Phlebotomy for CD4-count done at time of M-HTS, clients who retrieved results referred for HIV care	10	455	455	n retrieved CD4-count (within 90 days) *OR* initiated ART literacy (at any time)	N newly diagnosed	10% (45/455)	Not reported	Not reported	Not reported
**Chamie, 2012 [13]** Uganda, Rural	Multi-disease campaign held at community locations; POC CD4-count, Verbal referral	8	82	28	n attending at least one clinic appointment	N newly diagnosed	34% (25/64)	n initiated ART	N newly diagnosed & CD4 ≤100/cc^3^ and LTC	83% (5/6)
**Govindasamy,** **2013 [24]** South Africa, Urban & peri-urban	HTS provided five days per week at work sites (i.e. farms), outside various community locations; POC CD4-count, Written referral	6	294	Not reported	n attended HCF within ≤1mth if CD4≤200/cc^3^; ≤3mth if CD4 201-350/cc^3^; ≤6mth if CD4>350/cc^3^	N newly diagnosed CD4≤200/cc^3^	38% (18/48)	n on ART at 1mth follow-up	N newly diagnosed adults & CD4 ≤200/cc^3^and LTC	83% (15/18)
						N newly diagnosed CD4 201-350/cc^3^	53% (44/83)			
						N newly diagnosed CD4>350/cc^3^	53% (77/145)			
**Hatcher, 2012 [16]** Kenya, Urban	HTS in tents in six community sites; POC-CD4-count, Referral	Not reported	808	Not reported	n linked to care	N tested HIV+ and not in HIV care	10m: 81% (393/483)	Not reported	Not reported	Not reported
**Kranzer, 2012**^1^ ^**[**^^**25**^^**]**^ South Africa, Urban	HTS in a van parked at a township shopping centre/ front of a primary school; Up to 7 attempts to contact (by phone or in-person) if CD4 <350/cc^3^	11	102	Not reported	n linked to care	N newly diagnosed & CD4 ≤350/cc^3^	79% (26/33)	Not reported	Not reported	Not reported
**Labhardt, 2014 [11]** Lesotho, Rural	Community gatherings in villages followed by multi-disease services (within a cluster randomised trial); Referral only	8	75	Not reported	n linked to care	N newly diagnosed	25% (19/75)	Not reported	Not reported	Not reported
**Larson, 2012 [26]** South Africa, Setting Not reported	HTS in mobile units and tents/gazebos in taxi ranks/ shopping malls, POC CD4-count for some ^3^, Referral, Telephone FU	Not reported	Not reported	Not reported	n completed referral visit	N tested HIV+	54% (172/316)	Not reported	Not reported	Not reported
**van Zyl, 2015 [22]** South Africa Rural + urban	Mobile-HTS; Telephone FU	Not reported	Not reported	Not reported	n tested HIV+ and ART eligibility assessed.	N tested HIV+	51% (563/1096)	Not reported	Not reported	Not reported

*This proportion varied between studies with respect to whether it included individuals previously diagnosed and self-reporting HIV-positive status or only those who were newly diagnosed.

1. Incentives provided for study participation (not for linkage-to-care)

2. ART eligibility criteria not reported

3. If nurse providing M-HTS had equipment

A meta-analysis was considered but upon review of the data, not deemed appropriate for the following reasons: i) variability in definitions used for numerators and denominators when calculating proportions linked-to-care and initiated ART; ii) variability between studies in follow-up time and approaches for measuring time for linkage to care and treatment initiation. We have instead presented the relevant proportions (for linkage to care or ART initiation) in forest plots without summary estimates using Stata^TM^ version 15.0 for Windows (Stata-Corp, College Station, Texas).

## Results

### Characteristics of included studies

**Fig 1:**
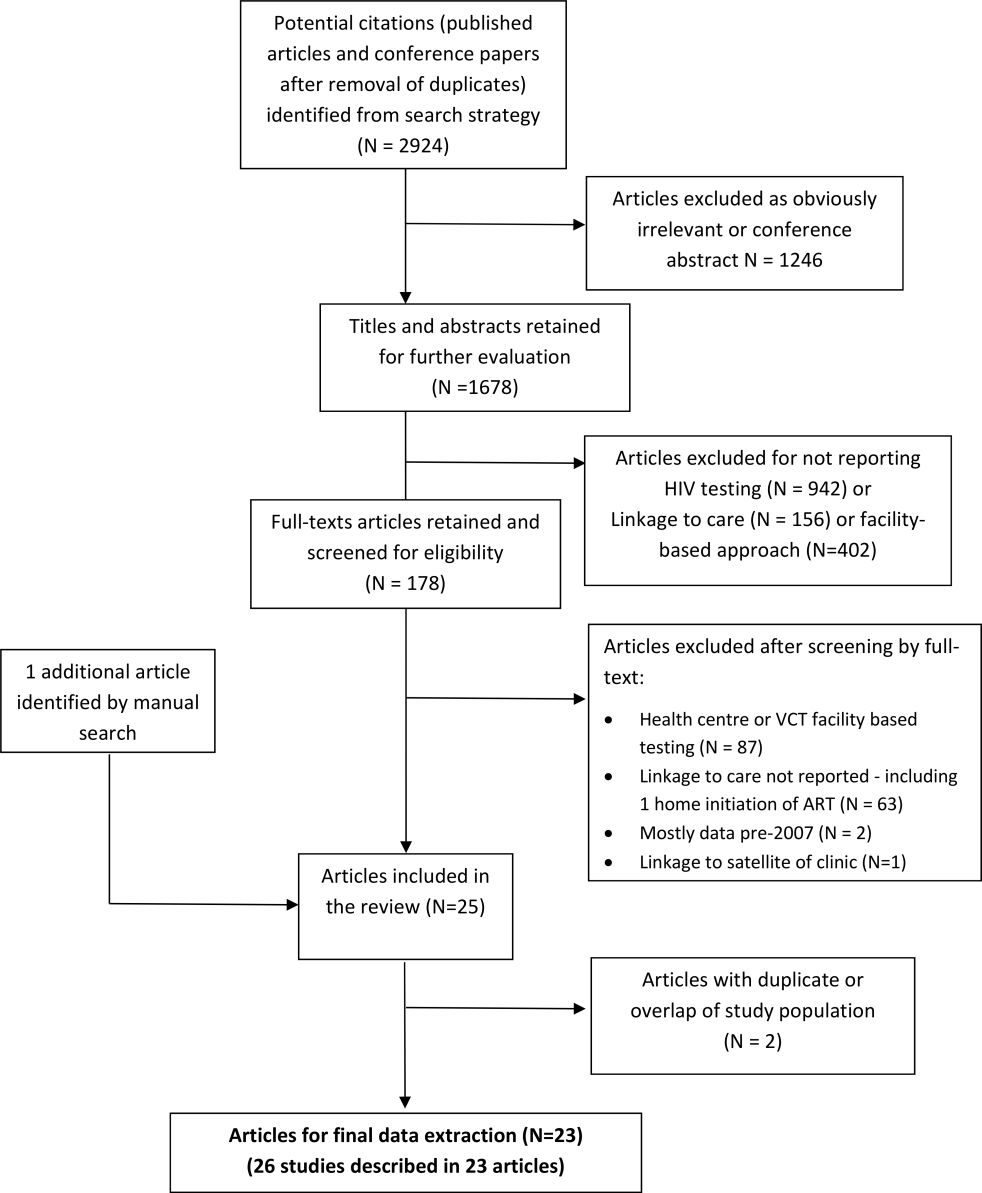
Flow diagram of study selection.

Our initial search yielded 2924 articles, of which 178 were reviewed as full-text articles and 23 were included in the final review (Fig 1). From these 23 articles we present results of analyses based on 25 “cascades” (Table 1) because one paper reported outcomes for HB- and CLB-HTS separately by modality, and a second reported results on random household HB-HTS and index TB patient household-member HB-HTS, as sub-groups. [11, 12] There were three community randomised trials and the remaining studies were observational cohorts. The studies were from six countries: Kenya, Lesotho, Malawi, South Africa, Swaziland, and Uganda [11–30], mostly from rural areas, and were conducted between 2008 and 2015. Most studies offered HTS for adults (mostly aged ≥18 years, but ≥13 years in one study), while 7 studies also offered HTS to children (mostly if they were orphaned or known to be HIV-exposed) (S1 Table). Regional adult HIV prevalence (obtained from UNAIDS national data if not reported by authors) ranged from 5–35%.

### Uptake, coverage and HIV-positivity among those tested

Fifteen cascades were on linkage to care after HIV detection through HB-HTS–most were door-to-door services provided by lay counsellors; two were targeted HB-HTS for household contacts of TB patients[12]; another was HB-HTS for randomly selected households[12]; and one study used oral self-test kits which were distributed by trained volunteers from the community.[18] One of the door-to-door HB-HTS studies was from a national HIV testing campaign.[17] Eight studies were on CLB-HTS approaches–which included use of mobile-vans, tents in busy community locations, shopping areas, transport hubs, etc. The two remaining cascades were on linkage outcomes from HB- and CLB-HTS in combination without stratifying linkage to care and ART outcomes by the approach of the HTS.[28, 30] Three of the twenty-five cascades provided HTS within a multi-disease intervention (one HB-HTS and two CLB-HTS cascades).[11, 13]

Twelve cascades were from articles which estimated population size eligible for the testing intervention (mostly HB-HTS cascades) and eighteen reported the number encountered and offered testing (S1 Table). Proportions offered testing among the population served by the HTS ranged from 61–98% for door-to-door HB-HTS cascades except in the article by Labhardt et al which reported 19% offered among those eligible. Proportions accepted testing among those offered testing (uptake of HTS) ranged from 35% to ~100% in the fifteen cascades in which this could be calculated. The proportion accepting HIV testing of the population eligible for the HTS (coverage of HTS) ranged from 17–91% (eight home based door-to-door HB-HTS cascades) while three CLB-HTS cascades reported coverage from 11–72%. Velen et al reported coverage among household contacts of TB index patients at 16%.

Detection of HIV-positivity among those tested ranged from 4–31% in HB-HTS cascades; 5–15% in CLB-HTS cascades (Table 1). In two articles which reported on both HB- and CLB-HTS in the same setting, HB-HTS had a slightly lower proportion detected with HIV than CLB-HTS (3.5% vs 4.7% and 3.6% vs 6.2%, respectively).[11, 30] Six cascades excluded individuals who self-reported knowing they were HIV-positive but the majority included previously known HIV-positive individuals among the number reported as detected by HTS—therefore proportions HIV-positive from those studies are not limited to newly diagnosed individuals.

As a result of losses from follow-up, data on proportions linked-to-care were limited to individuals who could be followed-up to identify linkage information (see Table 2). Twelve cascades relied on individual self-report of linkage/ART initiation (four of these used information on data which could be verified at clinics), eleven cascades used clinic records to obtain linkage and care outcomes, while two did not specify how outcomes were determined.

**Table 2:** Markers of study quality.

Author, Year	Participants offered HTS intervention	Did outcome exclude those already LTC (or on ART)?	How was outcome determined?	% in whom outcome *not* ascertained among those testing HIV+	Reasons outcome not ascertained	Period of Study for LTC (or ART initiation)	Timing of interim follow-up visits	Overall risk of bias in study
**Barnabas,** **2014** ^1^ **[29]**	Individuals consenting to door-to-door offer of HBHTS	Y (excluded individuals on ART)	Self-reported & review of clinic cards/medication with the individual	10% (n = 60/635) ^1^	Moved (57%; n = 34) Died (25%; n = 15) Withdrew (18%; n = 11)	12m	1, 3, 6, 9m with voice and/or text message reminders of follow-up visits	Low
**Dalal,** **2013 [14]**	Individuals consenting to a household visit from HBHTS & accepting an offer of HBHTS	Y	Self-reported	48% (n = 881/1839)	Not reported	1m	1m post-HTS	High
**Genberg,** **2015 [15]**	Individuals consenting to door-to-door offer of HBHTS	Y	Health facility records	2% (n = 33/1360)	LTFU (91%; n = 30) Died (9%; n = 3)	3m	Not reported	Low
**Iwuji, 2016 [31]**	Individuals consenting to door-to-door offer of HBHTS	Y	Health facility records	NA	NA	12m for LTC Within 3m of 1^st^ clinic visit for ART initiation	FU by phone or home visit after 3m if failed to LTC	Low
**Labhardt, 2014 [11]** **(HBHTS)**	Individuals consenting to door-to-door HBHTS	N	Health facility records	0	NA	1m	No FU visits	Low
**MacKellar,** **2016 [17]**	Individuals consenting to door-to-door offer of HBHTS	Y	Health facility records	Not reported ^2^	Not reported	26m	FU by telephone at 8w	Medium
**Maman, 2016 [32]**	Individuals consenting to door-to-door offer of HBHTS	Y	Health facility records	14% (n = 38/282)	Missing information on referral (52.6%; n = 20) Referred to private health facility or facility outside of district (47.4%; n = 18)	3m	No FU visits	Low
**Medley,** **2013 [19]**	Individuals consenting to door-to-door offer of HBHTS	N	Self-reported	32% (n = 350/1087)	Did not consent to FU visits (41%; n = 144) Migrated (25%; n = 89); Refused (20%; n = 70); Died (6%; n = 20); Missing/not at home (8%; n = 27)	2-4m post-HBHTS	3 attempts to visit home by HIV-positive peer educators	Medium
**Naik,** **2015 [27]**	Individuals consenting to door-to-door offer of HBHTS	Y	Self-reported & health facility records	18% (n = 79/438)	LTFU completely or LTFU prior to 3mth (90%; n = 71) Died (10%; n = 8)	3m	“Periodic” home visits or phone calls	Low
**Tumwebaze,** **2012 [20]**	Individuals consenting to door-to-door offer of HBHTS	N	Self-reported	2% (n = 3/152)	NR	3m	1 & 2m	Medium
**van Rooyen,** **2013 [21]**	Individuals consenting to door-to-door offer of HBHTS	N	Self-reported & review of care documentation/ medication with individual	4% (n = 5/137)	Died (60%; n = 3) Withdrew (40%; n = 2)	6m	1, 3, & 6m	Low
**MacPherson,** **2014 [18]**	Individuals opting to self-test (mostly at home)	Y (restricted to those not initiated on ART)	Health facility records	NA	NA	6m (ART initiation)	No FU visits	Medium
**Shapiro,** **2012 [12]** **(TB-contacts)**	HBHTS offered to household members of index TB patient	N	Not reported	Not reported	Not reported	2m (ART initiation)	Not reported	High
**Shapiro,** **2012 [12]** **(Random HH)**	HBHTS offered to household members of randomly selected households	N	Not reported	Not reported	Not reported	2m (ART initiation)	Not reported	High
**Velen,** **2016**	HBHTS offered to household members of index TB patients	Y	Self-reported	12% (n = 3/26)	Not reported	3m	Not reported	Medium
**Barnabas,** **2016 [28]**	Individuals consenting to door-to-door offer of HBHTS or self-selected through MHTS	Y (excluded individuals on ART)	Self-reported & review of clinic cards/medications with individual	3% (n = 40/1325) ^3^	Died (34%; n = 8) Moved (18%; n = 6) Withdrew (9%; n = 3) Unknown (68%; n = 23)	9m	1,3 and 6m for individuals randomised to lay counsellor FU	Low
**Parker, 2015 [30] (MHTS)**	Self-selection through MHTS	N	Health facility records	Not reported	Not reported	6m	Not reported	High
**Parker, 2015 [30] (HBHTS)**	Individuals consenting to door-to-door HBHTS							
**Bassett,** **2014 [23]**	Self-selection through MHTS	Y	Health facility records	Not reported	Not reported	3m	No FU visits	Medium
**Chamie,** **2012 [13]**	Self-selection through MHTS	Y	Not reported	22% (n = 18/82)	Implementation errors (72%; n = 13)	3m	Not reported	Medium
**Govindasamy** **2013 [24]**	Self-selection into mobile HTS	Y	Self-reported	6% (n = 18/294)	Refused (n = 4; 22%) Followed-up before follow-up period (n = 14; 78%)	Dependent on CD4 cell count–up to 6m	Telephone call 1w post-diagnosis	Low
**Hatcher,** **2012 [16]**	Self-selection through MHTS	Y	Self-reported	40% (n = 325/808)	Did not provide locator information (38%; n = 124) Not located at 10m FU (42%; n = 137) Did not consent to FU interview (15%; n = 47) Reported that they already enrolled in care prior to MHTS (5%; n = 17)	10m	FU visits conducted but timing NR	High
**Kranzer,** **2012 [25]**	Individuals accepting an invitation to MHTS	Y	Self-reported	20% (n = 8/41) (restricted to those with CD≤350)	Unable to contact by telephone or home visits (100%; n = 8)	1 & 3m (dependent on CD4 cell count at diagnosis)	Up to 7 attempts to contact (phone or face-to-face) individuals with CD4 ≤200 at 4w & CD201-350 at 12w post-HTS	Low
**Labhardt, 2014 [11]** **(MHTS)**	Self-selection through MHTS	N	Health facility records	0	NA	1m	No FU visits	Low
**Larson,** **2012 [26]**	Self-selection through MHTS	N	Self-reported	38% (n = 192/508)	Could not be contacted by telephone (100%; n = 192)	2m	Three attempts to contact individuals by phone 8w post-HTS	High
**van Zyl,** **2015 [22]**	Self-selection through MHTS	N	Self-reported	NA 4	NA	1m	Daily FU telephone calls	High

1 By 12m FU, LTFU reported among all individuals, including individuals on ART. Denominator therefore includes N = 254 known HIV+ & on ART

2 Not reported as outcome not reported separately for those detected through HB-HTS

3 Loss to follow-up is reported as individuals not followed-up at 9m; some of these individuals contributed to analysis of LTC and/or ART prior to being LTFU

4 Not applicable (NA): Individuals defined as “not linked to care” regardless of whether or not the individual was contactable. Among individuals not LTC, reasons available for N = 442: Asked not to be called (14%; n = 63); Deceased (0.2%; n = 1); Called many times (56%; n = 249) Incorrect information (18%; n = 79); No telephone (11%; n = 50)

### Linkage to care outcomes

#### Proportions linked-to-care

Definitions used for linkage to care varied as did methods of outcome ascertainment. Some studies described the outcome simply as “linkage to care” while others specified definitions used including proxy markers such as “CD4-count measured” or “CD4-count result received”; or identifying registration at the HCF where PLWH were referred (Table 1). Some studies restricted the denominator to newly diagnosed individuals when calculating proportions linked-to-care while others included those previously known to be HIV-positive provided they were not already in care/on ART. Seven studies did not report HIV-positive individuals as newly diagnosed or previously known PLWH and may have included individuals already in care.

**Fig 2:**
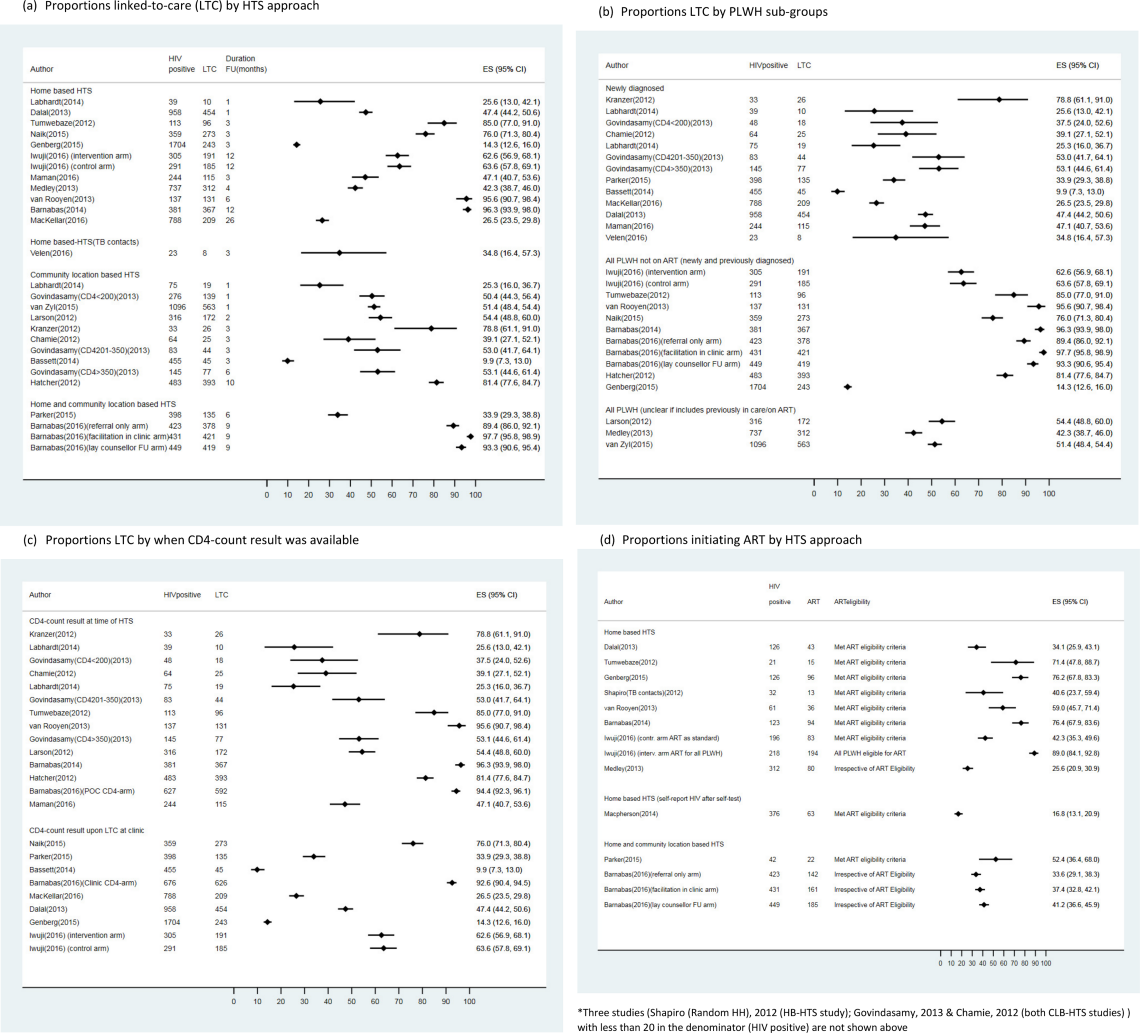
**a-d: Forest plots showing**:
**Proportions linked-to-care (LTC) by HTS approach (a)****Proportions LTC by PLWH sub-groups (b)****Proportions LTC by when CD4-count result was available (c)****Proportions initiating ART (among those eligible) by HTS approach (d)**

Proportions linked-to-care ranged from 14–96% among HB-HTS studies and from 10–79% among CLB-HTS studies over 1–12 months of observation, with no obvious differences by HTS approach (Fig 2A). Labhardt et al compared outcomes after HB- and CLB-HTS in the same setting and found no difference in proportions linked-to-care (HB-HTS: 26% (10/39) vs CLB-HTS: 25% (19/75)). The data suggest (see Fig 2B) that linkage to care was higher when all PLWH not already on treatment (newly diagnosed and previously known HIV-positive) were examined, than among newly diagnosed PLWH alone.

### Linkage to care by duration of follow-up

**Fig 3:**
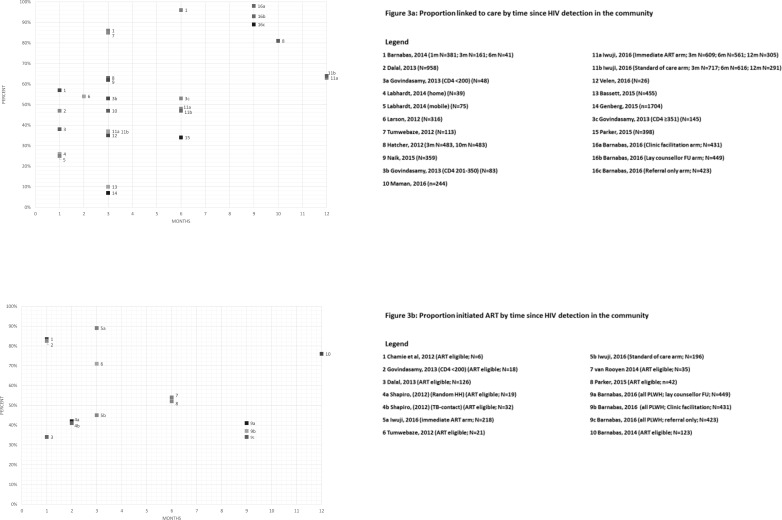
**a-b: Time taken following HIV detection at HTS for individuals**
**to LTC (a);****to initiate ART (b)**

The periods of observation varied between studies and time available for observing linkage varied as a result. Studies ascertained linkage outcomes by carrying out home-visits (occasionally in combination with telephone calls), once or at intervals after HTS; or consulted HCF records using a unique identifier to identify individuals who had been referred by HTS. The follow-up periods shown in Fig 3A represent the time between an individual being seen at HTS (when tested HIV-positive or self-reported HIV-positive status) and linkage-into-care. There was great variability in linkage to care between studies for similar time-points. The proportions linked-to-care ranged from 7–85% (Fig 3A) and the most commonly reported follow-up period for which linkage was reported was 3-months.

The total study periods are shown in Table 2. Few studies reported outcomes beyond 6-months following HTS (Fig 3A). Only three studies reported observed cumulative proportions linking-into-care over more than one time-point and while both showed progressive increases with time, the relative increase was not substantial.[16, 29] Six studies are not shown in Fig 3A because they did not report time taken for individuals to link. Some of these studies described overall proportions linked-to-care at various periods of time following the HTS programme, but not the time interval between an individual’s HIV detection at HTS and linkage to care. Eight studies presented cumulative probability of linkage to care curves over time, using time-to-event analyses. Those estimates suggest that most linkage appears to occur in the first 3-months—with some studies showing incremental benefit up to 12-months,[16, 17, 29, 34] while others showed plateauing over time after an initial steep increase in the first 1–3 months.[20, 21, 27, 32]

#### Approaches to facilitate linkage to care

Several studies used field-worker follow-up as a means to encourage and monitor linkage to care. Routine follow-up visits to PLWH were employed by 7 HB-HTS studies and 1 CLB-HTS study (Table 1). One randomised controlled trial (RCT) with a factorial design (reporting linkage from HB- and CLB-HTS in combination), examined three approaches following detection of HIV—follow-up visits by a lay counsellor in one study arm and facilitation by a lay counsellor in the clinic in another study arm, both to enhance linkage to care, compared with a standard-of-care referral only arm.[28] Both approaches to improve linkage achieved high linkage to care with the clinic facilitation arm achieving a stronger effect than the lay counsellor home follow-up, when compared to the control (referral only) arm. Two studies used telephone calls to routinely follow-up on PLWH detected through CLB-HTS.[22, 26] There is no clear evidence across all the studies that interventions to enhance linkage to care improved outcomes (S1A Fig)

Twelve studies provided CD4-counts at the time of HTS (using portable point-of-care (POC) technology or providing results within days of HTS if venous sampling was done for laboratory testing) (S1 Table). There is no clear evidence across studies that the proportion linked-to-care was higher if CD4-counts were provided at the time of HIV detection (Fig 2C). The above factorial design RCT also randomly allocated the clients from the 3 study arms described earlier to have either POC CD4-count or CD4-count sampling in the clinic. They found no benefit from POC CD4-count sampling over clinic testing for linkage to care, ART initiation or viral suppression.[28]

#### Predictors of linkage to care

Several studies reported factors associated with linkage to care. In ten studies (5 HB-HTS,4 CLB-HTS, 1 combined HB-CLB-HTS) which reported on potential gender differences, six reported that fewer men linked-to-care than women[15–17, 19, 22, 32] although one of those did not detect a statistically significant difference [19] and three other studies[13, 24, 30, 31] found no association between gender and linkage to care. However, the trend was always for fewer men than women to link. Six studies found that older adults were more likely to link-to-care[15–17, 27, 30, 32], while four observed no differences by age[13, 19, 24, 29]. Parker et al was the only study to consider linkage in adolescents specifically (defined as 9–19 years), and while they observed that this group appeared to be more likely to link-to-care the association was of borderline statistical significance (adjusted odd ratio of 2.5 (95% confidence interval: 1.0–6.0)).[30] Several other studies included people as young as 13-years of age but considered them as adults. Three studies described the association of education with linkage to care, and no clear pattern was observed.[15, 16, 24] Marital status was also not predictive of linkage to care.[13, 15, 19, 24, 32]

### ART initiation outcomes

#### Proportions initiating ART

Proportions initiating ART among those eligible were reported in nine HB-HTS, two CLB-HTS and both combined HB-/CLB-HTS cascades. As described above for linkage to care, the time available for ART initiation within the study periods varied (Table 2). The studies varied in ART eligibility criteria applied. Most studies had a CD4-count threshold of 350/cc^3^, while several had a threshold of 200-250/cc^3^ (Table 1). One study involved a community randomised trial (CRT) which examined the impact of universal treatment on HIV incidence and the intervention arm offered ART to all PLWH irrespective of CD4-count. Ascertainment of CD4-count eligibility for ART was done at the time of HTS in some studies but only upon linkage to care and sampling at the clinic in other studies (S1 Table).

Reported proportions initiating ART ranged from 23–93% in HB-HTS and combined HB-/CLB-HTS studies. In the above CRT, there was no notable difference in proportions initiating ART by CD4-count (87% among those with CD4-count >350/cc^3^ presenting to the clinic in the intervention arm vs 91–93% among those with CD4-count <350/cc^3^ in both arms).[31] The wide range in proportions initiating ART even among studies with comparable CD4-count thresholds is in part explained by the fact that the denominators varied. Most studies used HIV-positive individuals identified as eligible as the denominator while a minority used either all individuals identified as HIV-positive or those linked-to-care, and not already on ART (irrespective of CD4-count) (Table 1). Further, as shown in S1 Table some studies identified ART eligibility in the community at the time (or within days) of HTS, while in others eligibility was only assessed once individuals had linked-to-care. Both CLB-HTS studies had very small samples of PLWH (less than 20 individuals referred for ART) to assess initiation of ART (Table 1).[13, 24] There were no notable differences in reported ART initiation based on HTS approach (Fig 1D); newly diagnosed vs all PLWH not on treatment (new and previously diagnosed) (S1B Fig); and whether CD4-count results were provided during HTS (S1C Fig). Among PLWH who self-reported HIV-positive status after self-testing at home (and meeting ART eligibility criteria), 23% initiated ART.[18]

#### ART initiation by duration of follow-up

Eight studies reported ART initiation by time since HIV detection. There was no apparent trend and there was variability in the outcomes reported as seen in Fig 3B. Only two studies reported outcomes at more than one time-point,[16, 29] with one reporting cumulative outcomes based on time-to-event analysis estimates[29] (Fig 3B).

#### Predictors of ART initiation

Predictors of ART initiation were only examined in one study.[29] CD4-count was the only factor identified as predictive of ART initiation, with PLWH with CD4-counts <200/cc^3^ more likely to initiate ART than those with CD4-counts of 201-350/cc^3^. When we compared ART initiation by CD4-count threshold across the studies in this review there were no distinct differences notable (data not shown).

### Retention on ART and viral suppression

Retention on ART was reported by just one study. Macpherson et al reported that among those detected through self-testing at home, 6-months after ART initiation at the HCF, 76% (48/63) of participants were still on ART.[18] Four studies described viral suppression among participants on ART. One of them included patients who were already on ART before HB-HTS.[21] Two other studies both by Barnabas et al reported viral suppression (viral load < 1000 copies/ml) of 77% (59/77) among patients on ART for between 3–12 months in one study[29]; and 85% (412/483) of patients who initiated ART within 9-months of HIV detection in another study (with variable durations on ART).[28] The CRT by Iwuji et al reported no difference in viral suppression by ART initiation threshold (85% in both the intervention and control arms among treated individuals with median time on treatment of 265 days).[31]

## Discussion

Community-level HIV testing has become established as a feasible and effective approach to increasing knowledge of HIV status in SSA[35]. Others have published broad over-views of evidence following HTS (community and facility-based) and pooled outcomes, while acknowledging the limitations of summarising heterogeneous data.[2, 3] A recently published study focused its findings on linkage to care following home-based HTS.[36] In our systematic review, we aimed to cover multiple steps on the cascade of care yet provide detailed scrutiny—including examination of indicators used, measures of the numerators and denominators used to define linkage and treatment initiation, time-scales to observe outcomes etc.—with a specific focus on community-based approaches to HTS in SSA. We aimed to establish how effective community-level HTS approaches are at getting PLWH into care, beyond knowledge of HIV status alone, with a view to achieving 90-90-90 targets.

Definitions used for linkage to care and periods of observation for linkage to care and ART initiation outcomes varied between studies. The variability in denominators used in measuring ART initiation in particular, meant that outcomes were not in fact comparable. For instance, some studies included those who had previously linked to care (provided they were not on ART) in the denominator while others limited it to those who were newly detected. Given that to initiate ART PLWH had to link to care first (in all but the Macpherson et al home ART-initiation study)[18], one denominator involves one step in the cascade, while the other involves the cumulative proportion over two steps. Data were also limited to individuals who could be traced and the proportion of those identified HIV-positive at HTS in whom linkage and ART initiation outcomes could be ascertained was often low (Table 2). Most studies also relied on self-reported outcomes.

The above factors make summarising outcomes challenging and pooling of results potentially misleading. These important limitations in the data notwithstanding, we found that CLB-HTS and HB-HTS were equally effective at achieving LTC. We did not find discernible differences in terms of ART initiation although data on ART initiation after CLB-HTS were limited. There is a suggestion of higher linkage to care among those previously diagnosed (who had not already started ART) compared to newly diagnosed individuals. This fits with the idea that individuals need time to act on an HIV-positive diagnosis. However, this group is heterogenous and the barriers to link for those who have known their HIV status but not engaged with care compared to those already in care and have not started ART may be quite different.

As described, only one study performed a randomised comparison of interventions to enhance linkage and they reported nuanced findings.[28] While clinic facilitation by a lay counsellor was more effective than lay-counsellor home follow-up at increasing linkage to care, it is was the latter which was more effective at increasing uptake of ART (Table 1). There were also no differences in viral suppression at 9-months between PLWH randomised to intervention arms vs standard of care (referral for care only). This highlights the importance of measuring all the key steps of the cascade of care, as improvements in linkage to care may not translate to better treatment uptake or outcomes once on treatment. Nonetheless, the importance of optimising linkage was demonstrated by the CRT by Iwuji et al which showed that despite the availability of ART for all PLWH who were diagnosed through community-wide HB-HTS in the intervention arm (and high uptake among those who had linked), coverage of ART at a population level was undermined by the sub-optimal linkage to care.[34] Providing CD4-count results at the time of community-based HTS did not appear to influence linkage to care or ART initiation in our systematic review. The difference between our findings and most other data which have shown benefits following POC CD4-counts, is that we were looking at whether it benefited linkage to care following use in the community rather than use of POC CD4-counts in clinics for patients who had already attended.[37]

Studies reported that several patients were not initiated on ART (and told they were not eligible) despite being eligible.[30] [28] Transition to latest WHO guidelines of treatment for all PLWH will minimise decisions at the clinic level and reduce missed opportunities to offer/initiate treatment, provided that drugs are consistently available. Community delivery of ART for stable patients has to the potential to reduce the burden on HCFs and improve access for patients, thereby simplifying the cascade of care.[38] Macpherson et al examined home-initiation of ART following self-testing (in a randomised comparison with initiation of ART at the HCF which was included in this review).[18] They found higher proportions initiating ART in their home-initiation arm (although proportions retained on ART after 6 months among those who started were not different when compared with the facility-initiation arm). Subsequent same-day ART initiation trials including one with initiation at home upon diagnosis have also shown benefit across the cascade resulting in better viral suppression after 12m on treatment among those initiated on treatment on the same-day.[39–41]

Several studies on community-based HTS did not meet the eligibility criteria for inclusion in our review because data on linkage to care or ART initiation were not reported. This excluded some work-place or school-based HTS programmes and we could only include one study on a national testing campaign. Among eligible studies several of them stopped at reporting proportions linked-to-care without describing proportions initiating ART, especially CLB-HTS studies. The scant reporting on viral suppression is probably related to low access to routine viral load testing in most SSA settings, but only one study included data on retention among those started on ART yet data on this should be monitored and available to report.

The under-reporting within studies of multiple steps on the cascade alludes to the challenges in obtaining accurate data at the individual-level, for the continuum of care. In addition, it may indicate that health-care provider/researchers lack the resources to examine and report HIV care as a continuum, instead targeting efforts at individual steps in isolation.

The limitations of this review have been described at length above. The strengths however include the fact that we limited our search to studies conducted in SSA over the last decade, thereby maximising relevance for current practice. The attention to detail when examining definitions used to measure outcomes also sheds light on the complexity of the data presented in current literature. We made the deliberate choice not to summarise data from studies in our review using meta-analysis, given the heterogeneity in the data. Further, we provide a template of proposed standard indicators as a guide for data collection and reporting of community-based HTS programme performance on the cascade of care (S2 Fig). While not exhaustive, we hope that this will help minimise inconsistencies in future literature.

The UNAIDS 90-90-90 targets are an important reminder of the multiple steps needed to provide comprehensive HIV care. With currently published data it was not possible to estimate current performance against 90-90-90 goals. The premise of the 90-90-90 targets is that the total number of PLWH in a given setting has to be known or, more realistically, estimated accurately and only then can the first proportion be calculated (to compare against the first-90). There is ambiguity in the term “sustained ART” (in the definition of the second-90) or what duration should be allowed for viral suppression to be achieved (to compare against the third-90). The other challenge is that the UNAIDS targets are “point” measures—at any point of time, 90% of HIV-positive individuals need to know their status, 90% of those who know their status need to be “on ART”, and 90% of the latter need to be virally suppressed. Data on time to link-to-care or initiate ART are therefore difficult to use to estimate the coverage against the UNAIDS targets, as they are not point measures.

This systematic review has identified the gaps and inconsistencies in the current literature quantifying the continuum of care. We found no differences in linkage to care or ART initiation by community testing approach but comparisons were hampered by the variability in reporting. We recommend that standardised measures of reporting of steps of the cascade of care are much needed in order to be able to measure progress against targets and across settings.

## Supporting information

S1 Fig **Forest plots showing**:
**Proportions LTC by method of follow-up (S1a)****Proportions initiating ART by PLWH sub-groups (S1b)****Proportions initiating ART by when CD4-count result was available(S1c)**
(DOCX)

S2 Fig Proposed standard indicators for future reporting. (PDF)

S1 Table Additional study information. (DOCX)

S1 File PRISMA checklist. (PDF)

S2 File Search terms. (DOCX)
