# Supplementary material for: The cascade of care following community-based detection of HIV in sub-Saharan Africa – A systematic review with 90-90-90 targets in sight
Source: PLoS One. 2018 Jul 27;13(7):e0200737. doi: 10.1371/journal.pone.0200737 (PMC6063407; doi:10.1371/journal.pone.0200737)
Supplement: S1 Fig — Forest plots showing: Proportions LTC by method of follow-up (S1a)Proportions initiating ART by PLWH sub-groups (S1b)Proportions initiating ART by when CD4-count result was available(S1c) (DOCX) [file pone.0200737.s001.docx]

**Supplementary Figure 1: Forest plots showing (Supplementary 1a) LTC by method of follow-up (if any); (Supplementary 1b) ART initiation outcomes by PLWH sub-groups; (Supplementary 1c) ART initiation outcomes by when CD4-count result was available**

1. Proportions LTC by method of follow-up


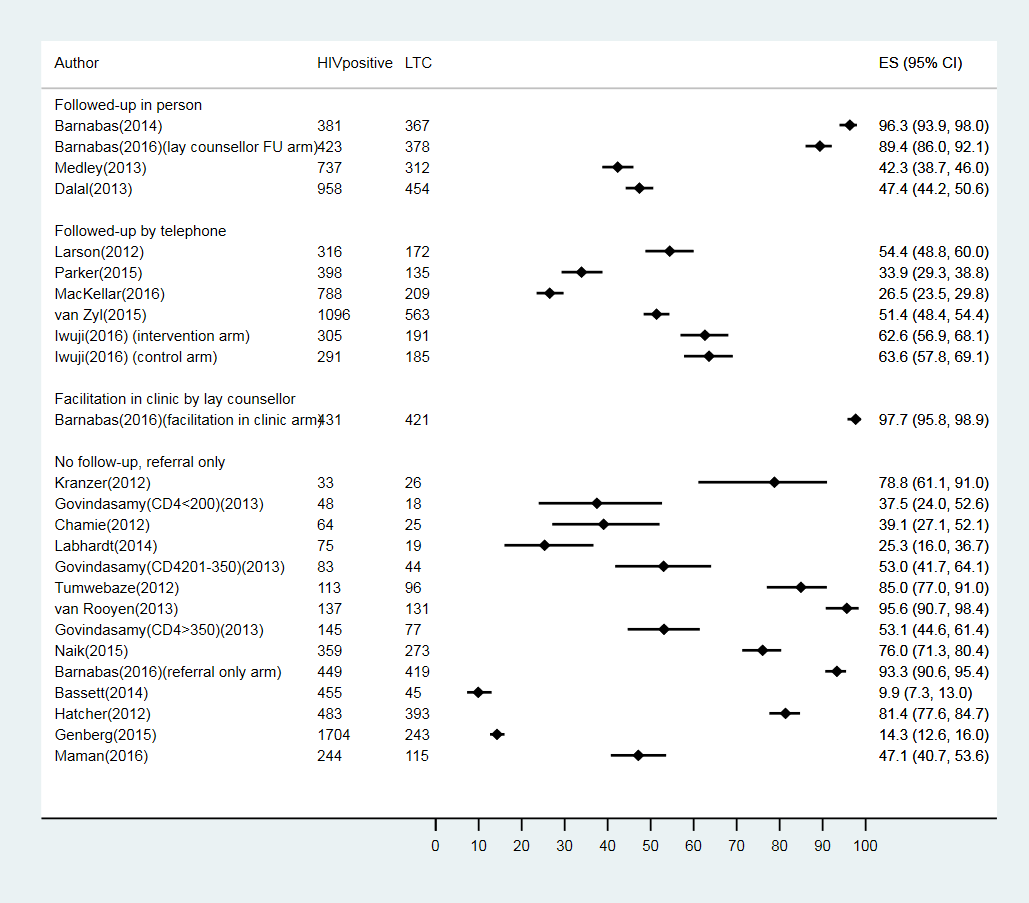


1. Proportions initiating ART by PLWH sub-groups


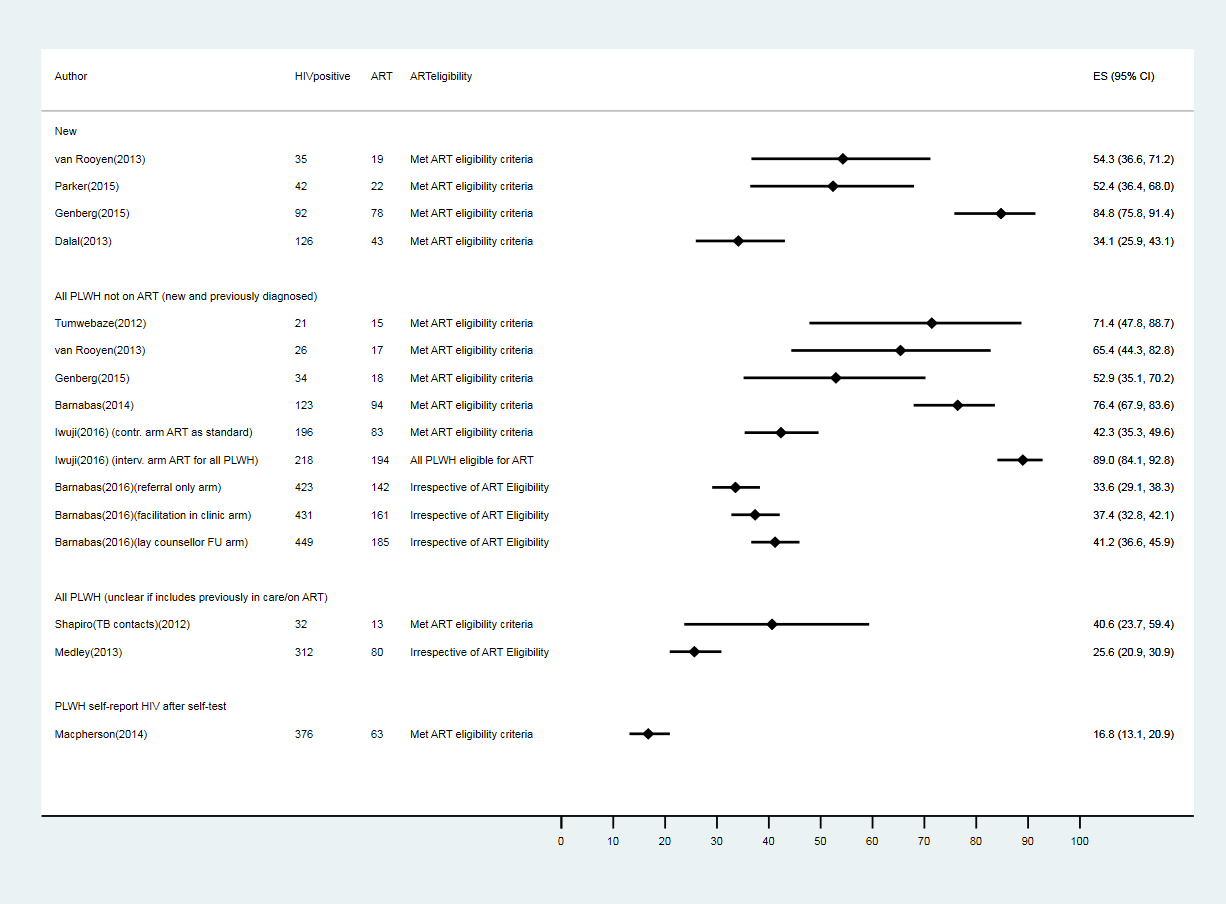


*Three studies (Shapiro (Random HH), 2012 (HB-HTS study); Govindasamy, 2013 & Chamie, 2012 (both CLB-HTS studies)) with less than 20 in the denominator (HIV positive) are not shown above

1. Proportions initiating ART by when CD4-count result was available


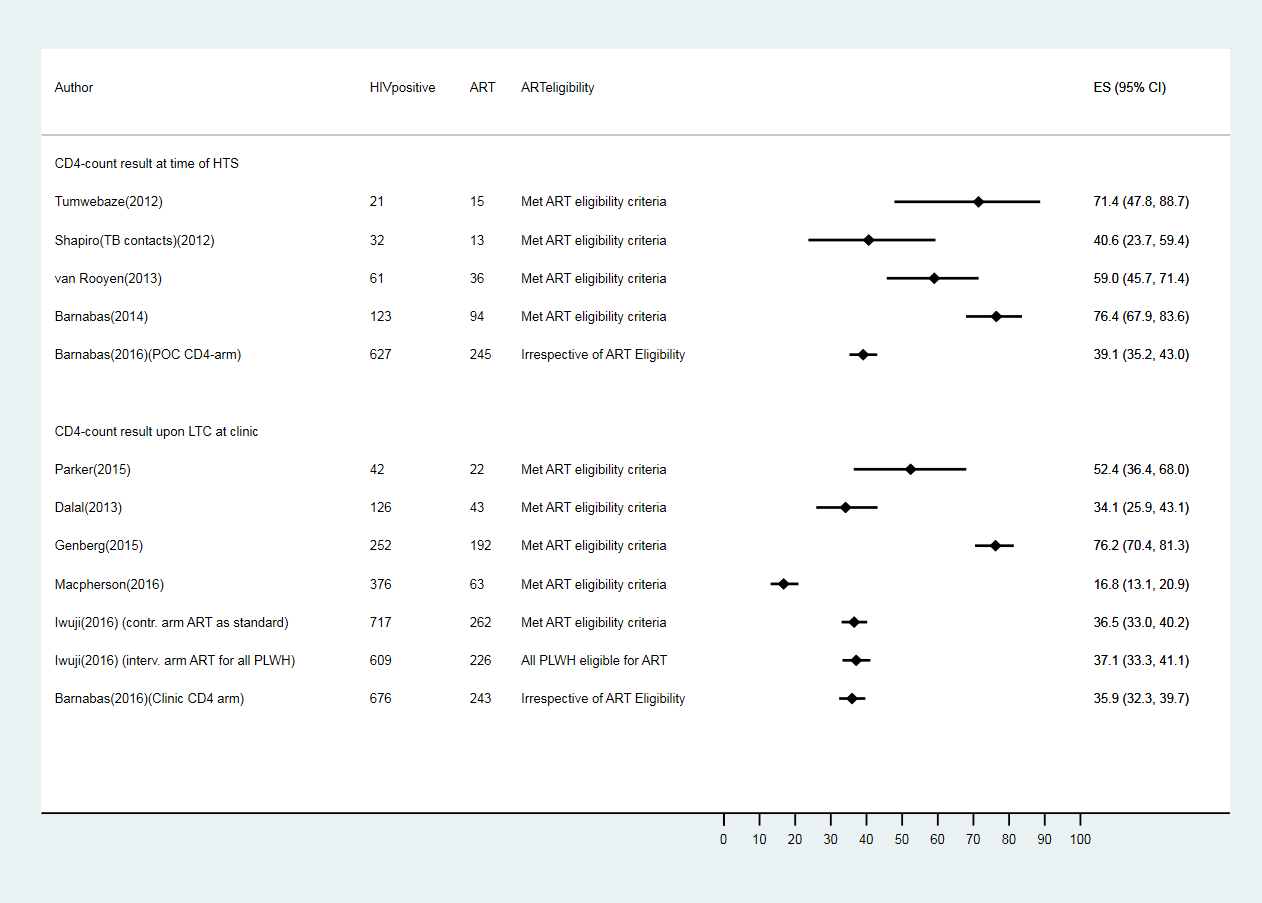


*Three studies (Shapiro (Random HH), 2012 (HB-HTS study); Govindasamy, 2013 & Chamie, 2012 (both CLB-HTS studies)) with less than 20 in the denominator (HIV positive) are not shown above
