# Supplementary material for: The cascade of care following community-based detection of HIV in sub-Saharan Africa – A systematic review with 90-90-90 targets in sight
Source: PLoS One. 2018 Jul 27;13(7):e0200737. doi: 10.1371/journal.pone.0200737 (PMC6063407; doi:10.1371/journal.pone.0200737)
Supplement: S2 Fig — (PDF) [file pone.0200737.s002.pdf]

Supplementary Figure-2: Template of standard indicators for reporting observed cascade of care data following community-based HTS (excludes "time-to-event" estimates)

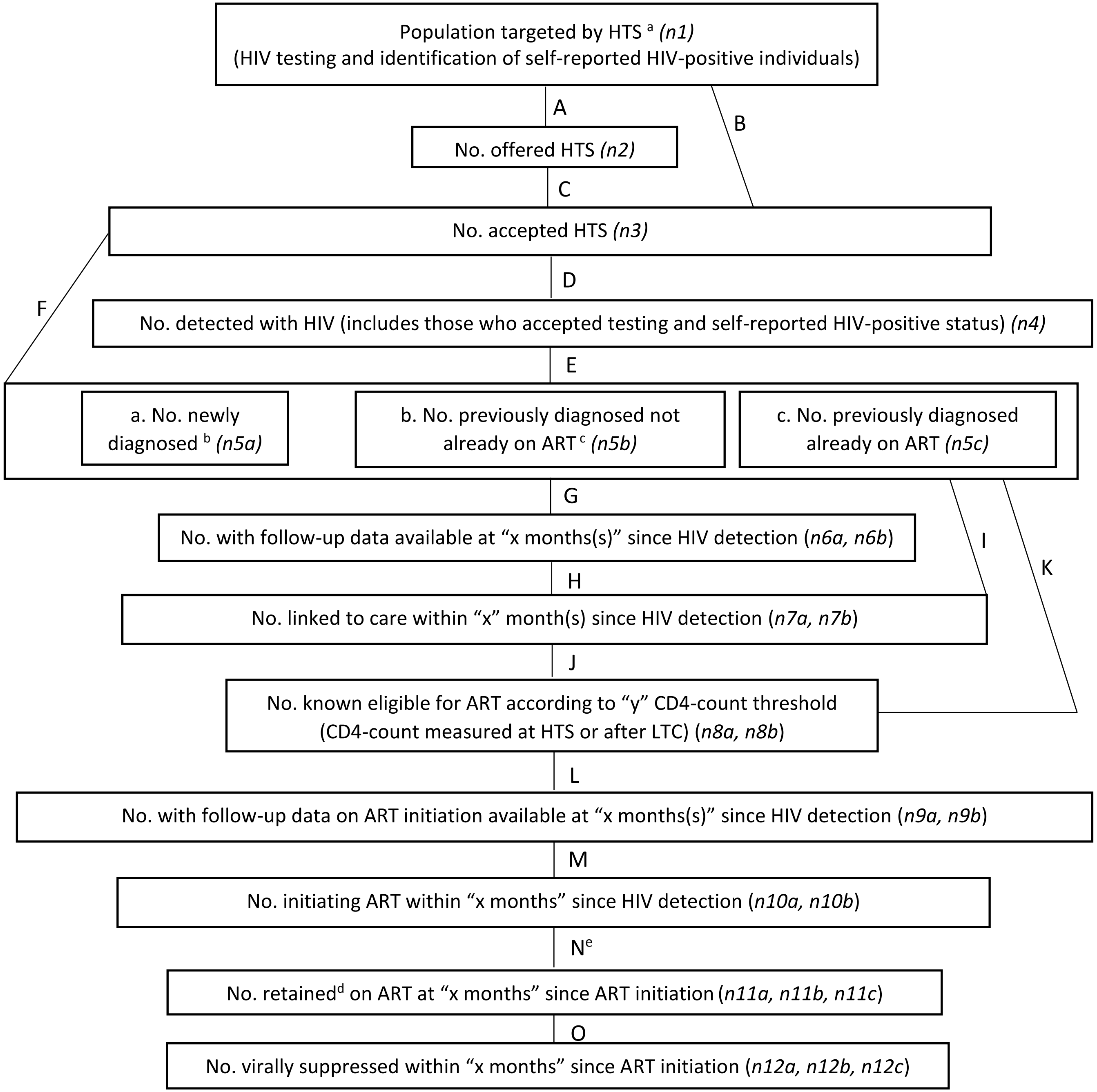

A. Proportion of target population offered HTS (*n2/n1*)
B. Population coverage of HTS (*n3/n1*)
C. Proportion accepted HTS among offered (*n3/n2*)
D. HIV-positivity in population accepting HTS (*n4/n3*)
E. Proportion of each HIV+ve sub-group (a.newly diagnosed; b. previously diagnosed not on ART; c.previously diagnosed already on ART) among those detected by HTS (*n5a/n4; n5b/n4; n5c/n4*)
F. Proportion of HIV+ve sub-group among those who accepted HTS (*n5a/n3; n5b/n3; n5c/n3*)
G. Proportion with follow-up data among respective sub-groups, at one or more of 1m / 3m / 6m / 12m (from when individual received HTS) [e] (*n6a/n5a; n6b/n5b*)
H. Proportion linked to care among those with follow-up data by respective sub-groups, at one or more of 1m / 3m / 6m / 12m (*n7a/n6a; n7b/n6b*)
I. Proportion known linked to care among all those detected with HIV by respective sub-groups, at one or more of 1m / 3m / 6m / 12m (*n7a/n5a & n7b/n5b*)
J. Proportion identified as eligible for ART initiation (100% if universal treatment applies) among those LTC by respective sub-groups, at one or more of 1m / 3m / 6m /12m (*n8a/n7a & n8b/n7b*)
K. Proportion identified as eligible for ART initiation (100% if universal treatment applies) among those who accepted HTS by respective sub-groups, at time of detection (*n8a/n5a & n8b/n5b)*
L. Proportion on whom there is follow-up data on ART initiation, among those eligible (100% if universal treatment applies) by respective sub-group (*n9a/n8a & n9b/n8b*)
M. Proportion initiating ART among those eligible (100% if universal treatment applies) with follow-up data, by sub-group (*n10a/n9a & n10b/n9b*)
N. Proportion documented as retained on ART, by sub-group (*n11a/n10a, n11b/n10b, n11c/n5c*[e])
O. Proportion virally suppressed among those retained on ART, by sub-group (*n12a/n11a, n12b/n11b, n12c/n11c*)

a. Estimated or measured eg by census of target community *b. Not self-reporting as HIV-positive* *c. This group could be further sub-divided into already in care (& not on ART) vs not already in care (& not on ART)*
d. Retained = data confirming "on ART" in last 3 months *e. For those previously started on ART – denominator is n5c and time since ART was first initiated*
