# Supplementary material for: The cascade of care following community-based detection of HIV in sub-Saharan Africa – A systematic review with 90-90-90 targets in sight
Source: PLoS One. 2018 Jul 27;13(7):e0200737. doi: 10.1371/journal.pone.0200737 (PMC6063407; doi:10.1371/journal.pone.0200737)
Supplement: S1 Table — (DOCX) [file pone.0200737.s003.docx]

**Supplementary table 1: Additional study information**

| **Author,**  **Year** | **Primary objective** | **Estimated population eligible for testing intervention** | **Number offered testing**  **(%) ^1^** | **Number accepted testing (%) ^2^** | **Adults or children offered HTC** | **% of individuals accepting HTS that are male ^3^** | **Median CD4-count measured**  **at diagnosis**  **/cc^3^ (IQR)** | **Median CD4-count measured**  **at LTC**  **/cc^3^ (IQR)** | **CD4-count result available when**  **1. HIV detected (or within days)**  **2. after LTC** |
| --- | --- | --- | --- | --- | --- | --- | --- | --- | --- |
| **HB-HTS** | | | | | | | | | |
| Barnabas, 2014 | To assess linkage to local HIV clinics, ART initiation among individuals eligible by national guidelines and viral suppression at 12mths | Not reported | 3545 | 3393  (96%) | Adults aged ≥18y | 42%  (n=1424/3393) | 456  (288-628) | Not reported | 1 |
| Dalal,  2013 | To assess acceptance of HB-HTS, HIV prevalence and treatment referral rates | Not reported | 24450 | 19966  (82%) | Adults & children^4^ | 45%  (n=8889/19966) | 403  (252–594) | Not reported | 2 |
| Genberg,  2015 | Proportion with known HIV infection identified at HB-HTS who had ever engaged in care; time to and predictors of linkage to care (LTC) | 66723 (Including children – ineligible in this study) | 32333  Adults (≥13y) | 32269 (~100%) | Adults (≥13y) | Not reported | Not reported | 436  (267-558) | 2 |
| Iwuji,  2016 | To evaluate process indicators within a cluster-randomised trial in rural KwaZulu-Natal which was evaluating whether immediate ART in HIV-positive individuals could significantly reduce HIV incidence  at the population level. | 12894 | 9927  (77%) | 8233  (83%) | Adults  (≥16y) | 31%  (2567/8233) | Not reported | Not reported | 2 |
| Labhardt, 2014  (HB-HTS) | To compare HTS uptake, HIV prevalence and LTC within 1mth between HB-HTS and mobileCLB-HTS | 6311 | 1171  (19%) | 1083  (92%) | Adults & children | Men (≥12yrs):  30% (n=247/812) | 438 (265-650) | Not reported | 1 |
| MacKellar, 2016 | To evaluate compliance with new national linkage and retention standard operational procedures and enrolment in HIV care | Not reported | Not reported | Not reported | Adults | Not reported | Not reported | 280  (165–420) | 2 |
| Maman,  2016 | To examine HIV status awareness at  population level; factors associated  with HIV-positive status awareness prior to the survey and subsequent LTC | 8277 | 7634  (92%) | 7270  (95%) | Adults  (≥15y) | 41%  (2995/7270) | 328 (men)/  440 (women) | Not reported | 1 |
| Medley,  2013 | To describe HB-HTS coverage and HIV prevalence, & characteristics associated with enrolment into HIV clinical care within two to four months after receiving an HIV diagnosis during HB-HTS among adult residents of a longitudinal Health and Demographic Surveillance System | 15933 | 12035  (76%) | 9895  (82%) | Adults & children^4^  (≥13y) | Not reported | Not reported | Not reported | Not reported |
| Naik,  2015 | To determine what proportion of adults LTC after HB-HTS within 3mths & the factors associated with LTC | Not reported | Not reported | Not reported | Adults  (≥14y) | Not reported | Not reported | 341  (224-542) | 2 |
| Tumwebaze, 2012 | To evaluate HB-HTS combined with an electronic triage platform as a platform to facilitate LTC | 1941 | 1587  (82%) | 1558  (98%) | Adults (≥18y) | 47%  (n=724/1558) | 479  (330–715) | Not reported | 1 |
| van Rooyen, 2014 | To assess whether HB-HTS with POC CD4 & facilitated referrals to HIV care achieved: high HTS coverage; identified HIV-positive individuals unaware of their HIV status; reduced potential barriers to care engagement. & reduced infectiousness through high uptake of & adherence to ART | 739 | 726  (98%) | 671  (92%) | Adults (≥18yr) | 33%  (n=222/671) | 435  (301–591) | Not reported | 1 |
| Shapiro, 2012  (non TB-contact households) | To determine the prevalence of undiagnosed TB & HIV in households of index TB-patients, to compare the yield of TB & HIV in TB-contact households with randomly selected non TB-contact households & determine the efficiency of targeting households  of patients with TB for active case-finding interventions | Not reported | 2843 | 1568  (55%) | Adults & children^4^  (excl <5y) | Not reported | 381  (236–567) | Not reported | 1 |
| Shapiro, 2012  (TB-contact households) |  |  | 983 | 521  (53%) | Adults & children^4^  (excl <5y) |  | 383  (285–561) | Not reported | 1 |
| Velen,  2016 | To evaluate HCT uptake during household contact tracing among contacts of index TB patients; factors associated with the uptake of testing and LTC | 1887 | 876  (46%) | 304  (35%) | Adults  ((≥14yr) | 36%  (109/304) | Not reported | Not reported | Not reported |
| MacPherson, 2014  (HIVST at home) | To investigate whether offering optional home initiation of ART after HIVST might increase population level ART uptake compared with HIVST combined with facility-based initiation | 8466 | Unknown (Test kits made available for 8466) | Unknown (Participant choice to disclose) | Adults (≥16y) | Not reported | Not reported | 187  (100-256) | 2 |
| **CLB- and HB-HTS** | | | | | | | | | |
| Barnabas, 2016 | To assess whether community-based HTS with counsellor support and POC CD4 increases uptake of ART compared to HB-HTS | Not reported | 15700 | 15332  (98%) | Adults (≥16y) | 43%  (n=6533/15332) | POC arm:  486  (344-653) | Clinic arm & could recall CD4: 512  (384-670) | 1 & 2  (based on randomisation arm) |
| Parker, 2015  (HB-HTS) | To evaluate the feasibility, yield & LTC of two strategies: HB-HTS and CLB-HTS | 12269 | 7484  (61%) | 7026  (94%) | Adults & children^4^  (excl <5y) | 39%  (n=3106/7026) | Not reported | Not reported | 2 |
| Parker, 2015  (CLB-HTS) |  | 18207 | Not reported | 2034 | Adults & children^4^  (excl <5y) | Not reported |  |  | 2 |
| **CLB-HTS** | | | | | | | | | |
| Bassett,  2014 | To evaluate yield and LTC from CLB-HTS compared to clinic based HTS in high prevalence South African township | Not reported | Not reported | 4703 | Adults (≥15y) | 46%  (2163/4703) | 416  (287-587) | Not reported | 2 |
| Chamie,  2012 | To test the feasibility and diagnostic yield of integrating NCD and other communicable disease services into a rapid, high through-put, community based HIV testing and referral campaign for all residents of a rural Ugandan parish;  and to determine rates and predictors of post-campaign LTC by disease | 6300  (3150 adults) | 4343  (2323 adults (74%)) | 4108  (2282 adults  (98%)) | Adults & children^4^ | Not reported ^5^ | Adults:  415  (281-568) | Not reported | 1 |
| Govindasamy, 2013 | To determine the yield of newly-diagnosed HIV, TB symptoms, diabetes & hypertension from a M-HTS unit, & assess CD4 testing, LTC & correlates of LTC & barriers to care | Not reported | 9806 | Not reported | Adults  ( ≥18y) | Not reported | *Mean: 481*  *(95% CI:*  *458-505)* | Not reported | 1 |
| Hatcher,  2012 | To assess predictors of LTC among individuals testing HIV-positive after community-based HTS | Not reported | Not reported | 10203 | Adults (≥18y) | Not reported | Not reported | Not reported | 1 |
| Kranzer,  2012 | To compare yields of newly diagnosed HIV & advanced HIV between individuals attending CLB-HTS as participants in a population-based HIV seroprevalence survey & those accessing the service for routine HTS | Not reported | 1300 (number recruited ie given flyer about CLB-HTS) | 936 (included in LTC analysis) | Adults | Recruited HTS: 52% (n=491/936)  Client-initiated HTS (not given flyer): 56% (n=488/877) | Recruited HTS: 385  (267–602)  Client-initiated HTS: 415 (309-680) | Not reported | [1](http://www.samj.org.za/index.php/samj/article/view/4162/2975) |
| Labhardt, 2014  (CLB-HTS) | To compare HTS uptake, HIV prevalence and LTC within 1mth between HBHTS and CLB-HTS | 4909 | 1392  (28%) | 1207  (87%) | Adults & children^4^ | Men (≥12yrs):  24% (n=236/994) | 400 (207-629) | Not reported | 1 |
| Larson,  2012 | To assess the proportion of patients LTC within 8wks of CLB-HTS under routine conditions & the impact of  including POC CD4 testing on the proportion of patients LTC within  8wks of CLB-HTS | Not reported | Not reported | Not reported | Adults (≥18y) | Not reported | Among individuals offered POC CD4: 414 (251-589) | Not reported | 1 |
| van Zyl,  2015 | To determine LTC & time to LTC after CLB-HTS combined with a call centre for facilitating LTC | Not reported | Not reported | Not reported | Not reported | Not reported | 370 (IQR Not reported) | Not reported | Not reported |

1. Percentage shown in brackets refers to proportion of population of offered testing among population eligible for HTS – if numerator and denominator reported in given study.
2. Proportion accepted testing among those offered HTS – if numerator and denominator reported.
3. Three studies also reported % of all men offered HTS who accepted testing: Dalal, 2013 - 80% (n=8889/11068); Labhardt, 2014 (HB-HTS) - 94%; (n=247/262); Labhardt, 2014 (CLB-HTS) - 97%; (n=236/243)
4. Children whose biological mother was deceased or known to be HIV+
5. Authors did not report% of males accepted but reported % of male residents who attended (were offered HTS) = 52% men (vs 95% of women)
