# Supplementary material for: The cascade of care following community-based detection of HIV in sub-Saharan Africa – A systematic review with 90-90-90 targets in sight
Source: PLoS One. 2018 Jul 27;13(7):e0200737. doi: 10.1371/journal.pone.0200737 (PMC6063407; doi:10.1371/journal.pone.0200737)
Supplement: S2 File — (DOCX) [file pone.0200737.s005.docx]

**Search terms for “The cascade-of-care following community-based detection of HIV –**

**a systematic review with 90-90-90 targets in sight”**

1. HIV

HIV Infections[MeSH] OR HIV[MeSH] OR hiv[tw] OR hiv-1*[tw] OR hiv-2*[tw] OR hiv1[tw] OR hiv2[tw] OR hiv infect*[tw] OR human immunodeficiency virus[tw] OR human immune deficiency virus[tw] OR human immuno-deficiency virus[tw] OR human immune-deficiency virus[tw] OR ((human immun*) AND (deficiency virus[tw])) OR acquired immunodeficiency syndrome[tw] OR acquired immunodeficiency syndrome[tw] OR acquired immunodeficiency syndrome[tw] OR acquired immune-deficiency syndrome[tw] OR ((acquired immun*) AND (deficiency syndrome[tw])) OR ”sexually transmitted diseases, viral“[MESH:NoExp]

1. Home based

Home-based or home based or homebased OR door to door OR door-to-door OR home care services OR homecare services OR homecare OR home care OR home-care OR home access OR home OR in-home OR domicile

1. Community

Community* OR community based OR community-based OR mobile*

1. Work place

Work place OR work-place OR workplace OR work OR occupation*

1. School- based

School-based OR school OR school*

1. VCT

Voluntary Counselling or voluntary Counseling or voluntary Testing or hiv testing or Vct or hbvct or hct

1. Africa

sub-saharan Africa OR south Africa OR Africa South of the Sahara OR Lesotho OR Swaziland OR Namibia OR Botswana OR Zimbabwe OR Mozambique OR Malawi OR Zambia OR Angola OR Tanzania OR Rwanda OR Burundi OR Democratic republic of congo OR Republic of congo OR Uganda OR Kenya OR Ethiopia OR Somalia OR Sudan OR Central african republic OR Cameroon OR Gabon OR Guinea OR Chad OR Nigeria OR Niger OR Togo OR Benin OR Ghana OR Burkina faso OR Cote d'ivoire OR Ivory coast OR Liberia OR sierra leone OR Senegal OR Gambia

1. (1 AND 5) AND (2 OR 3 OR 4 OR 5)
2. 8 AND 7
3. Limit 7 to 01/01/2006 to 31/05/2017
